# Ameliorative Effect of Beetroot Against Folic Acid Deficiency: A Review

**DOI:** 10.1002/fsn3.71417

**Published:** 2026-01-02

**Authors:** Amara Rasheed, Farhan Saeed, Ali Ikram, Muhammad Tayyab Arshad, Sammra Maqsood, Feroza Naveed, Mariam Islam, Emmanuel Laryea

**Affiliations:** ^1^ Department of Food Science Government College University Faisalabad Faisalabad Pakistan; ^2^ University Institute of Food Science and Technology The University of Lahore Lahore Pakistan; ^3^ Functional Food and Nutrition Program, Center of Excellence in Functional Foods and Gastronomy, Faculty of Agro‐Industry Prince of Songkla University Hatyai, Songkhla Thailand; ^4^ Department of Food Science and Technology Kwame Nkrumah University of Science and Technology Kumasi Ghana

**Keywords:** anemia, anti‐inflammatory, antioxidant, folate, fruit, healthcare

## Abstract

A lack of vegetables or other folate‐rich foods in the diet leads to folate insufficiency. The most significant cause of this malformation may be a lack of folic acid. Folic acid deficiency, a critical maternal health component, can result in megaloblastic anemia, neural tube anomalies, cardiovascular complications, cancer, and Deoxyribonucleic acid (DNA) imbalance. Beetroot ( *Beta vulgaris* ) is one of the most widely consumed vegetables worldwide. Red beets have attracted more attention because of their rich source of bioactive substances, including betalain, indolent nitrates, polyphenols, and folates, as well as minerals and vitamins found within the tuberous root. Red beets show strong potential for usage as functional ingredients in the food and healthcare industries due to their numerous health‐promoting qualities. The plant has antioxidative and anti‐inflammatory properties that may be useful in the treatment of a variety of disorders.

## Graphical abstract

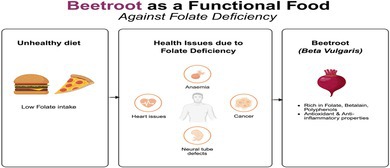

## 1 Introduction

Folate is a broad term that comprises both the naturally occurring folates present in foods and folic acid, the synthetic form present in fortified foods and supplements. Folate, commonly known as vitamin B9 or folacin, is a water‐soluble B‐vitamin (Naderi and House 2018). Natural folates occur in diverse chemical forms and exist in both dietary and physiologically active states within the human body. However, folic acid is the most widely used synthetic form in food fortification and vitamin supplements. Folic acid has no physiological role until converted into active folates, and since the body cannot produce this vitamin, it must be obtained through diet or supplements (Shulpekova et al. 2021). Historically, the dietary folate requirement was defined as the amount needed to prevent deficiency severe enough to cause clinical symptoms, such as anemia. Recently, the recommended dietary allowance (RDA) was determined based on achieving sufficient red blood cells (RBCs) or folate concentrations without abnormal hematologic markers (Fenech 2012). Red blood cell folate has also been demonstrated to link with folate reserves of the liver and is utilized as a long‐term folate indication. Plasma folate is an unreliable indicator of folate status because it only reflects recent dietary intake rather than long‐term folate levels (Devalia et al. 2014). The dietary folate equivalent (DFE) approach was developed to account for the bioavailability differences among synthetic folic acid and the natural forms of folate found in foods. One microgram of dietary folate is equal to one DFE whereas one microgram of folic acid is equivalent to 1.7 DFE. The explanation for the disparity is that at least 85% of folic acid is absorbed when introduced to foods or consumed as a dietary supplement, although only around 50% of the folate organically found in the food is absorbed (Scott et al. 2000). Folic acid (vitamin B9) is an essential dietary micronutrient required for DNA synthesis, methylation, and red blood cell production (Ebara 2017). Folic acid deficiency is associated with a higher risk of megaloblastic anemia, neural tube defects in developing fetuses, and elevated homocysteine levels, which are connected with cardiovascular diseases (Kancherla and Black 2018; Chrysant and Chrysant 2018). Although synthetic folic acid supplements are widely recommended, consuming folate‐rich foods with natural bioactive compounds may offer additional health benefits (Ali et al. 2022). A highly nutritious vegetable, beetroot contains antioxidants, dietary nitrates, and betalains, which have been shown to enhance metabolic well‐being, suppress oxidative stress, and enhance vascular function (Clifford et al. 2015; Chen et al. 2021). Emerging evidence suggests that beetroot may help mitigate folic acid deficiency‐related conditions. The bioactive phytochemicals in beetroot, i.e., betacyanins and betaxanthins, possess antioxidant and anti‐inflammatory properties that may complement folate metabolism; beetroot is a relatively good source of folate (Hadipour et al. 2020; Sadowska‐Bartosz and Bartosz 2021). As per studies by Krajka‐Kuźniak et al. (2013), betalains can potentially enhance the antioxidant defense of cells by enhancing the activity of pathways controlled by nuclear factor erythroid 2‐related factor 2 (Nrf‐2). This may be protective against oxidative DNA damage due to folate deficiency. As per studies (Bahrami et al. 2021; Mirmiran et al. 2020), beetroot dietary nitrates augment endothelial function and nitric oxide bioavailability, which may decrease cardiovascular hazards associated with hyperhomocysteinemia, a common outcome of not having enough folate. Furthermore, beetroot has also been revealed through research to help shield liver and kidney tissues against oxidative stress that can intensify in the event of folate deficiency (Albrahim 2020; Krajka‐Kuźniak et al. 2012). Because certain bacteria in the digestive system aid in the natural synthesis of folate, its potential to impact the gut microbiota may have consequences on folate biosynthesis (Bationo et al. 2020). Beets, as well as folic acid supplements, provide a possible nutritional intervention for nutritional insufficiency, and this is especially true in functional foods. Offering a holistic explanation of beetroot's medicinal properties, this paper explores the means by which its bioactive constituents can counteract the adverse effects of folic acid deficiency.

## 2 Roles of Folic Acid in the Body

In general, FA participates in a variety of bodily functions, such as the metabolism of amino acids, nucleic acids, and one‐carbon molecules.

### 2.1 One‐Carbon Metabolism

The transfer of one‐carbon units is a key function of folate cofactors in the body. Folate cofactors participate in the metabolic processes of amino or nucleic acids by acting as recipients and donors of carbon units (Kennedy 2016). Parental nutritional imbalances and unhealthy lifestyles can disrupt several metabolic pathways, with one‐carbon metabolism being the most critical. One‐carbon metabolism is involved in many physiological activities, including purine and thymidine synthesis, methionine, glycine, and serine balance, and epigenetic changes and redox reactions regulation. Defects of one‐carbon metabolism can cause a range of consequences at numerous stages of development, such as nonalcoholic fatty liver, cancers, vascular disease, cognitive decline, and dementia. Vitamin B9 (folate) functions as a coenzyme within the one‐carbon unit transferase family and is involved in the transmission of one carbon units. During pregnancy, folate deficiency has been strongly associated with several fetal abnormalities, including encephalopathy, spina bifida, and encephalocele (O'Kane and Begg 2019).

### 2.2 Nucleic Acid Metabolism

**FIGURE 1:**
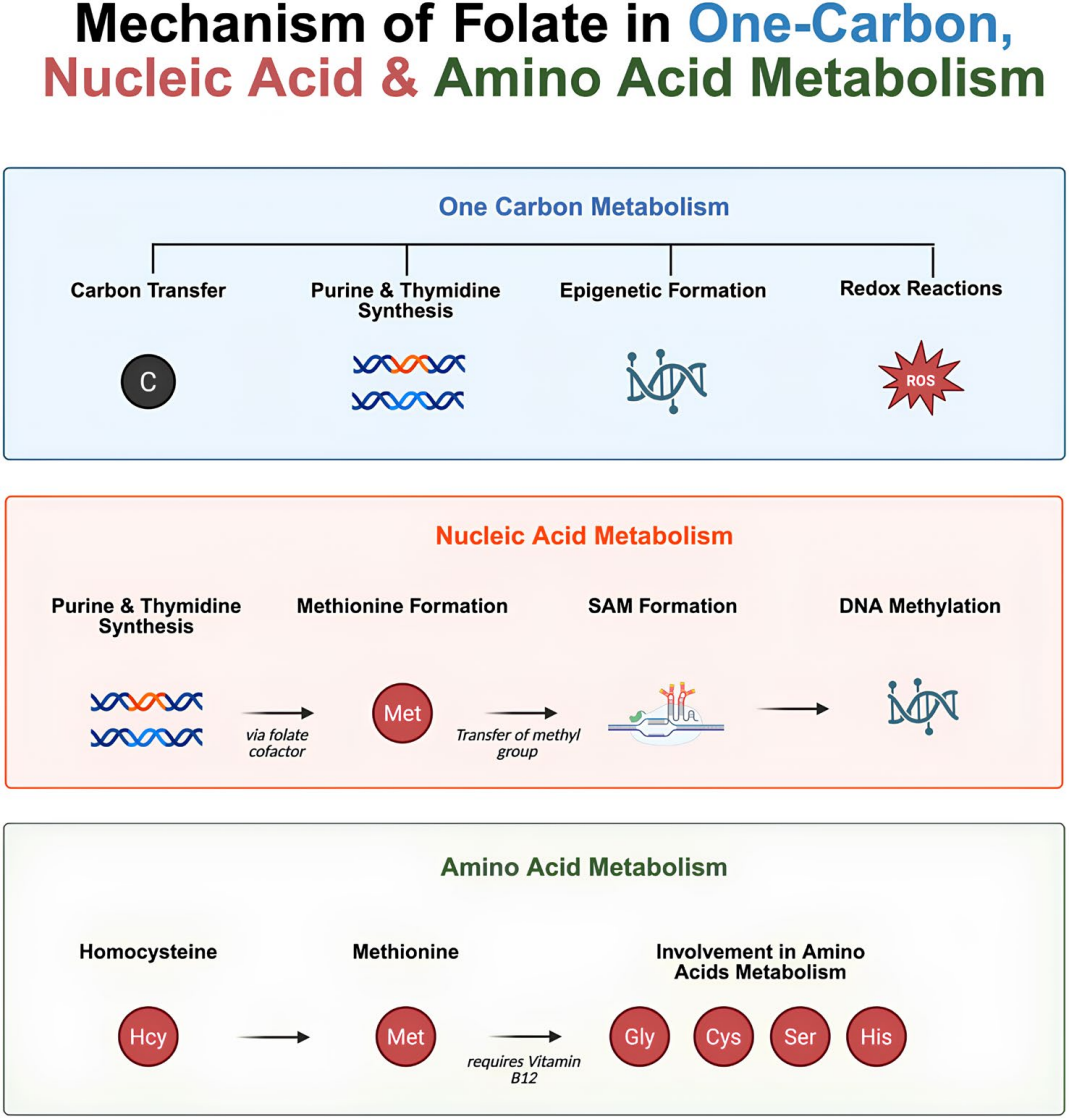
Mechanism of folate in one‐carbon, nucleic acid, and amino acids metabolism.

Folate coenzymes contribute significantly to the metabolism of DNA via two distinct mechanisms. First, they are required for the synthesis of purines and thymidine, which serve as essential substrates for DNA biosynthesis. Second, folate cofactors are necessary for the conversion of homocysteine to methionine, which in turn is required for the formation of S‐adenosylmethionine (SAM). SAM acts as a universal methyl donor in numerous biological processes including the methylation of proteins, phospholipids, RNA, and DNA. Methylation of DNA plays a role in regulating gene expression and is essential for cell development (Abbasi et al. 2018). A detailed mechanism of folate involvement in one‐carbon metabolism, nucleic acid synthesis, and amino acid metabolism is illustrated in Figure 1.

### 2.3 Amino Acid Metabolism

Methionine, glycine, cysteine, serine, and histidine are among the amino acids that require folate coenzymes for metabolism. Methionine synthase catalyzes the development of methionine from homocysteine, an enzyme that requires both folate (as 5‐methyltetrahydrofolate) and vitamin B12 (Ebara 2017). Folic acid deficiency can lead to a decrease in methionine synthesis and an upsurge in homocysteine levels. The levels of homocysteine in the blood are widely recognized as a risk factor for a variety of chronic disorders, particularly heart diseases and cognitive impairment (Vidmar et al. 2019).

## 3 Health Risks of Folic Acid Deficiency

Human folic acid deficiency has been associated with megaloblastic anemia, neonatal neural tube abnormalities, and heart problems. The demand for folic acid increases during pregnancy, making it essential for both maternal health and fetal development. Folate is also related to the development of cancer, particularly colorectal cancer. Folic acid, on the other hand, is required for regular DNA synthesis and repair (Catala et al. 2019). Folate deficiency can lead to chromosome breakage, uracil misincorporation, and an imbalance in DNA precursors. Since the human body cannot synthesize this vitamin, it must be obtained through diet and supplementation (LeBlanc et al. 2018).

**FIGURE 2:**
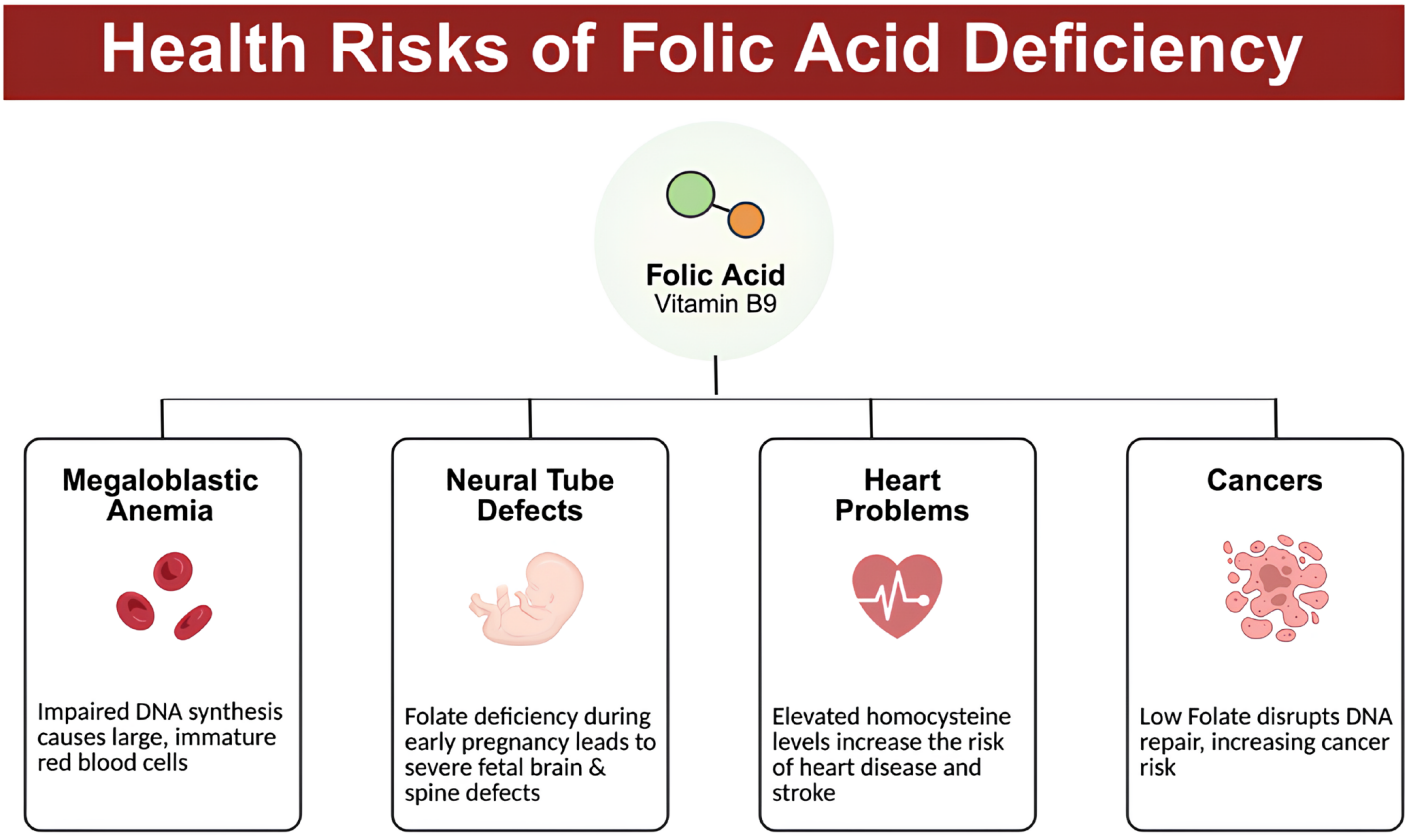
Health risks of folic acid deficiency.

Folate deficiency during pregnancy is a well‐recognized risk factor for neural tube defects (NTDs). Folate deficiency increases the risk of NTDs and contributes to hyper‐homocystinemia, a condition linked to an elevated risk of cardiovascular disease and NTDs (Moussa et al. 2016). Folic acid deficiency can impair fetal development and nutrition, and it is also considered a potential risk factor for cognitive decline and disability in older adults (Ali et al. 2022). Folic acid deficiency has been linked to cognitive decline in the elderly as shown in Figure 2, largely due to inadequate intake of vitamin B9 or its poor absorption, metabolism, or retention.

### 3.1 Megaloblastic Anemia

Megaloblastic anemia is a type of macrocytic anemia marked by the existence of massive red cell precursors called megaloblasts in the bone marrow as a result of a DNA deficiency that leads to asymmetrical erythroblast development. Hypovitaminosis, notably folate and cobalamin deficits, is the most common cause of megaloblastic anemia (Ndhlovu 2016).

These vitamins are required for DNA synthesis. Folic acid deficiency may result from inadequate intake due to factors such as alcoholism, malnutrition, poverty, or restrictive diets, and is particularly common in older or hospitalized patients. An increased requirement during pregnancy, hemolysis, or malabsorption conditions such as tropical sprue, celiac disease, jejunal resection, or Crohn's disease can also lead to folic acid deficiency. Anticonvulsants and chemotherapeutic drugs can sometimes produce megaloblastic anemia due to a deficiency of folic acid by interfering with its metabolism (Kovalyova et al. 2021).

### 3.2 Neural Tube Defects (NTDS)

Widespread cell division characterizes fetal growth and development. Folate is necessary to produce DNA or RNA. NTDs are a group of heterogeneous disorders that affect the development of the fetal central nervous system between 21 and 28 days after fertilization. Brain malformations (like anencephaly or encephalocele) and spinal cord malformations (like spina bifida) are a few of the tragic and even lethal malformations that comprise the general term “NTDs” (van Gool et al. 2018).

There are numerous variables, such as heredity, congenital malformations, and environmental exposure, which all collectively form the intricate and multifaceted etiology of NTDs. However, detailed health risks associated with folic acid deficiency are summarized in Table 1. Conversely, Bibbins‐Domingo et al. (2017) found that women who consumed higher amounts of folic acid before pregnancy had a significantly lower risk of neural tube defects in their infants. With an incidence ranging from 1 to 10 per 1000 births, depending on geography and ethnicity, neural tube defects are among the most common congenital malformations. For women who have previously given birth to an affected child, the recurrence risk is estimated at 2%–5% (Kancherla and Black 2018).

**TABLE 1:** Health risks associated with folic acid deficiency.

Health risk	Mechanism/effects	Population at risk	Preventive measures	References
Megaloblastic anemia	Impaired DNA synthesis in erythroblasts → enlarged, immature RBCs (megaloblasts)	Pregnant women, elderly, malnourished individuals	Folic acid supplementation (400–800 μg/day); dietary folate (leafy greens, legumes)	Devalia et al. (2014), Ebara (2017)
Neural tube defects (NTDs)	Folate‐dependent neural tube closure failure in embryos (e.g., spina bifida, anencephaly)	Pregnant women, especially in early gestation	Periconceptional folic acid (400 μg/day); fortified foods (e.g., cereals)	Bibbins‐Domingo et al. (2017), Kancherla and Black (2018)
Hyperhomocysteinemia & cardiovascular disease	Folate deficiency ↑ homocysteine → endothelial dysfunction, oxidative stress, thrombosis	Elderly, CKD patients, individuals with poor folate intake	Folic acid (0.5–5 mg/day) + B12; beetroot (nitrates ↓ blood pressure)	Chrysant and Chrysant (2018), Bahrami et al. (2021)
Increased cancer risk	Folate deficiency → DNA hypomethylation, chromosomal breaks (↑ colorectal, breast cancer risk)	IBD patients, alcoholics, low‐folate diets	Folate‐rich diets (beetroot, lentils); caution: excess folate may promote tumor growth	Catala et al. (2019), Moazzen et al. (2018)
Cognitive decline	Elevated homocysteine → neurotoxicity, ↑ Alzheimer's/dementia risk	Elderly, vitamin B12‐deficient individuals	Combined B9/B12 supplementation; dietary nitrates (beetroot juice)	Kennedy (2016), Kelly et al. (2013)
Pregnancy complications	Folate deficiency → preterm birth, fetal growth restriction	Pregnant women in low‐resource settings	Prenatal folic acid (600 μg/day); fortified staple foods	Bationo et al. (2020), Moussa et al. (2016)
Immune dysfunction	Folate vital for lymphocyte proliferation → weakened immunity	Malnourished children, HIV/AIDS patients	Balanced diet with folate + zinc/iron; beetroot (immunomodulatory betalains)	Das et al. (2017), Hadipour et al. (2020)
Depression & mental health	Folate regulates neurotransmitter synthesis (serotonin, dopamine)	Patients with depression, alcoholism	Methylfolate supplements; folate‐rich foods (spinach, beetroot)	Kennedy (2016), Ali et al. (2022)
Osteoporosis	Hyperhomocysteinemia → bone resorption ↑, collagen impairment	Postmenopausal women, elderly	Folic acid + B12; beetroot (antioxidants ↓ oxidative bone loss)	Ravimannan and Nisansala (2017)
Liver disease	Folate deficiency → fatty liver, ↑ oxidative stress (NASH progression)	Alcoholics, obese individuals	Beetroot juice (betalains ↓ liver enzymes, protect hepatocytes)	Krajka‐Kuźniak et al. (2012), Albrahim (2020)

Based on Viswanathan et al. (2017) and other investigations, the US Public Health Service concluded that pregnant women must consume 400 mg of folic acid per day to decrease the occurrence of NTDs. In addition, it was recommended that women with a history of pregnancies affected by NTD consume 4 mg of folic acid per day. Pregnant women who took folic acid supplements along with a balanced diet experienced a 60%–100% lower risk of neural tube defects (NTDs) compared to those who did not (Kondo et al. 2017).

### 3.3 Hyperhomocysteinemia and Heart Problems

One important co‐substrate in this process is 5‐methyltetrahydrofolate, the active form of folate required by methionine synthase to convert homocysteine into methionine. A lack of dietary folate leads to the accumulation of homocysteine in the body. The breakdown of one‐carbon compounds produces homocysteine as an intermediate, which can promote cell proliferation, oxidative stress, inflammation, thrombosis, and endothelial dysfunction. Elevated homocysteine levels (mild to moderate hyperhomocysteinemia) are strongly associated with atherosclerotic vascular diseases, including coronary artery disease and stroke, as confirmed by several clinical studies (Cianciolo et al. 2017).

Cardiovascular diseases (CVDs) are responsible for the overwhelming majority of deaths worldwide, based on the World Health Organization. According to the World Health Organization (WHO), CVDs encompass coronary artery disease, cerebrovascular and arterial diseases, pulmonary embolism, congenital heart defects, and deep vein thrombosis, all of which affect the cardiovascular system. One of the main pathophysiological components of arterial CVDs is atherosclerosis, which cardiovascular risk factors exacerbate. These factors include smoking, high cholesterol levels, abdominal obesity, diabetes, high blood pressure, and psychological consequences (Chrysant and Chrysant 2018).

Most, but not all, cases of arterial CVDs can be explained by traditional risk factors. Additional risk factors, such as a rise in homocysteine concentrations, can cause different types of vascular injury, which demands the enactment of additional tests. There has been a lot of research on the mechanism through which homocysteine may increase the risk of heart disease. However, it is possible that this process involves the detrimental effects of homocysteine on blood coagulation, artery dilatation as well as the thickness of artery walls (Li et al. 2016).

### 3.4 Cancers

It is possible that low consumption of folic acid contributes to gene mutations and chromosome breakage, which are often characteristics of cancer development. This is because folate is essential for methylation, synthesis of DNA and RNA, and other processes. Folate deficiency leads to nucleotide shortage, which results in DNA mutation and genomic instability (Moazzen et al. 2018). Genome maintenance depends greatly on DNA replication and repair. A reduction in 5,10‐methyl THF may inhibit the thymidylate synthase enzyme from catalyzing the conversion of deoxy‐uridine monophosphate (dUMP) to deoxythymidine monophosphate (dTMP), resulting in the accumulation of uracil and the depletion of thymine. If uracil is mistakenly incorporated during DNA replication or repair, it can lead to DNA damage in the form of strand breaks and point mutations (Burr et al. 2017).

Folate could be protective against certain cancers, have minimal to no effect against others, and even create an increased risk in the long term based on how much is consumed. Higher folate intake has been associated with a nearly 50% lower risk of squamous cell carcinoma of the head and neck, a 35% reduced risk of oral cavity and pharyngeal cancers, a 41% lower risk of all histological types of esophageal cancer, a 34% reduction in pancreatic cancer, and a 16% reduction in bladder cancer compared to lower folate intake (Hijaz et al. 2016).

## 4 Assessment of Folate Status

A population's folic acid status can be evaluated using a diversity of measurement techniques, comprising the concentration of folate in serum or RBCs, urinary folic acid catabolites like para‐aminobenzoyl glutamate and para‐acetamido benzoyl glutamate, or dietary intakes of vitamin recorded with the help of food frequency questionnaires or the quantitative 24‐h dietary recall method (Bationo et al. 2020).

The World Health Organization recommends using red blood cell folate concentrations of 400 ng/mL or 906 nmol/L as an indicator for detecting folate deficiency and preventing neural tube defects in reproducing women, based on serum concentrations of 3 ng/mL or 6.8 nmol/L (Shulpekova et al. 2021).

## 5 Prevention of Folate Deficiency

Because the human body does not have a biochemical mechanism that can produce folate, it must be obtained exclusively through the consumption of foods that contain it. Folate is extremely difficult to store, and a deficiency can appear anywhere from a few weeks to a few months later in individuals whose diets are weak in folate (Bationo et al. 2020).

In addition, diets for many pregnant women and children are often inadequately supplemented due to the low bioavailability or loss of cooking nutrients, thereby reducing folate intake. Folate consumption is further inadequate because of poor bioavailability and loss during cooking. There are a variety of ways to achieve the daily dose of folate that is necessary for the successful prevention of folate deficits (Harika et al. 2017). These include consuming a more varied diet, consuming folic acid that has been fortified into foods, or taking folic acid supplements. Taproot and beetroot refer to the same part of the beet plant. In North America, the beet plant is most commonly referred to as the beet, but it is also called table beet, garden beet, red beet, and golden beet. It is one of the several *B. vulgaris* cultivars farmed for the edible taproots and the leaves, sometimes known as beet greens (Sood and Gupta 2017).

The Romans were responsible for the cultivation of several of the modern‐day veggies, including beetroot. When the researchers discovered in the 19th century that sugar could be made from beets, this led to a significant increase in the commodity's market worth. The United States, France, America, Poland, Russia, and Germany are currently the top five commercial producers worldwide. The culinary traditions of central and eastern Europe are home to a wide variety of traditional beetroot dishes, including the world‐famous soup known as borscht made with beetroot (Pandita et al. 2020).

## 6 Nutritional Composition of Beetroots

Table 2 presents the nutritional composition of beetroot (per 100 g fresh weight), accentuating its rich profile as a major root vegetable with a unique combination of nutrients. These nutrients include proteins, sugars, carbs, vitamins, particularly the B class and vitamin C, minerals, and fiber (Septembre‐Malaterre et al. 2018). They have a significant polyphenol content, betalains, and antioxidants such as coumarins, beta‐carotene, lutein, sesquiterpenes, triterpenes, and flavonoids like astragalin, tiliroside, rhamnocitrin, kaempferol, and rhamnetin that are known to have a variety of positive effects on one's health (Chhikara et al. 2019).

**TABLE 2:** Nutritional composition of beetroot (per 100 g fresh weight).

Nutrient	Value	Part analyzed	References
Proximate composition			
Water	87.5%	Tuber	Mudgal et al. (2022)
Energy	43 kcal	Tuber	Mudgal et al. (2022)
Protein	1.35–1.89 g	Tuber	Ceclu and Nistor (2020), Mudgal et al. (2022)
Fat	0.15–0.30 g	Tuber	Ceclu and Nistor (2020), Mudgal et al. (2022)
Carbohydrates	7.23–9.56 g	Tuber	Ceclu and Nistor (2020), Mudgal et al. (2022)
Dietary fiber	1.9–3.25 g	Tuber	Ceclu and Nistor (2020), Mudgal et al. (2022)
Ash	1.08–1.4 g	Tuber	Ceclu and Nistor (2020), Mudgal et al. (2022)
Vitamins			
Vitamin C	4.9–30 mg	Tuber & leaves	Mudgal et al. (2022), Ceclu and Nistor (2020)
Folate (folic acid)	109 μg	Tuber	Mudgal et al. (2022)
Vitamin B6	0.067–0.106 mg	Tuber & leaves	Mudgal et al. (2022)
Niacin	0.334–0.400 mg	Tuber & leaves	Mudgal et al. (2022)
Vitamin A (IU)	36–6326 IU	Tuber & leaves	Mudgal et al. (2022)
Minerals			
Potassium (K)	30.12–762 mg	Tuber & leaves	Kale et al. (2018), Mudgal et al. (2022)
Sodium (Na)	72.58–226 mg	Tuber & leaves	Kale et al. (2018), Mudgal et al. (2022)
Calcium (Ca)	12.20–117 mg	Tuber & leaves	Kale et al. (2018), Mudgal et al. (2022)
Iron (Fe)	0.75–0.80 mg	Tuber	Kale et al. (2018), Mudgal et al. (2022)
Magnesium (Mg)	23–70 mg	Tuber & leaves	Mudgal et al. (2022)
Zinc (Zn)	0.21–0.38 mg	Tuber & leaves	Kale et al. (2018), Mudgal et al. (2022)
Manganese (Mn)	0.359–0.79 mg	Tuber & leaves	Kale et al. (2018), Mudgal et al. (2022)
Bioactive compounds			
Betaine	128.7 μg	Tuber	Mudgal et al. (2022)
Betalains (betalain content varies by cultivar)	0.5–1.2 g/kg	Tuber	Clifford et al. (2015)
Nitrates	250–500 mg	Tuber	Bahrami et al. (2021)

## 7 Bioactive Substances in Beetroot

**FIGURE 3:**
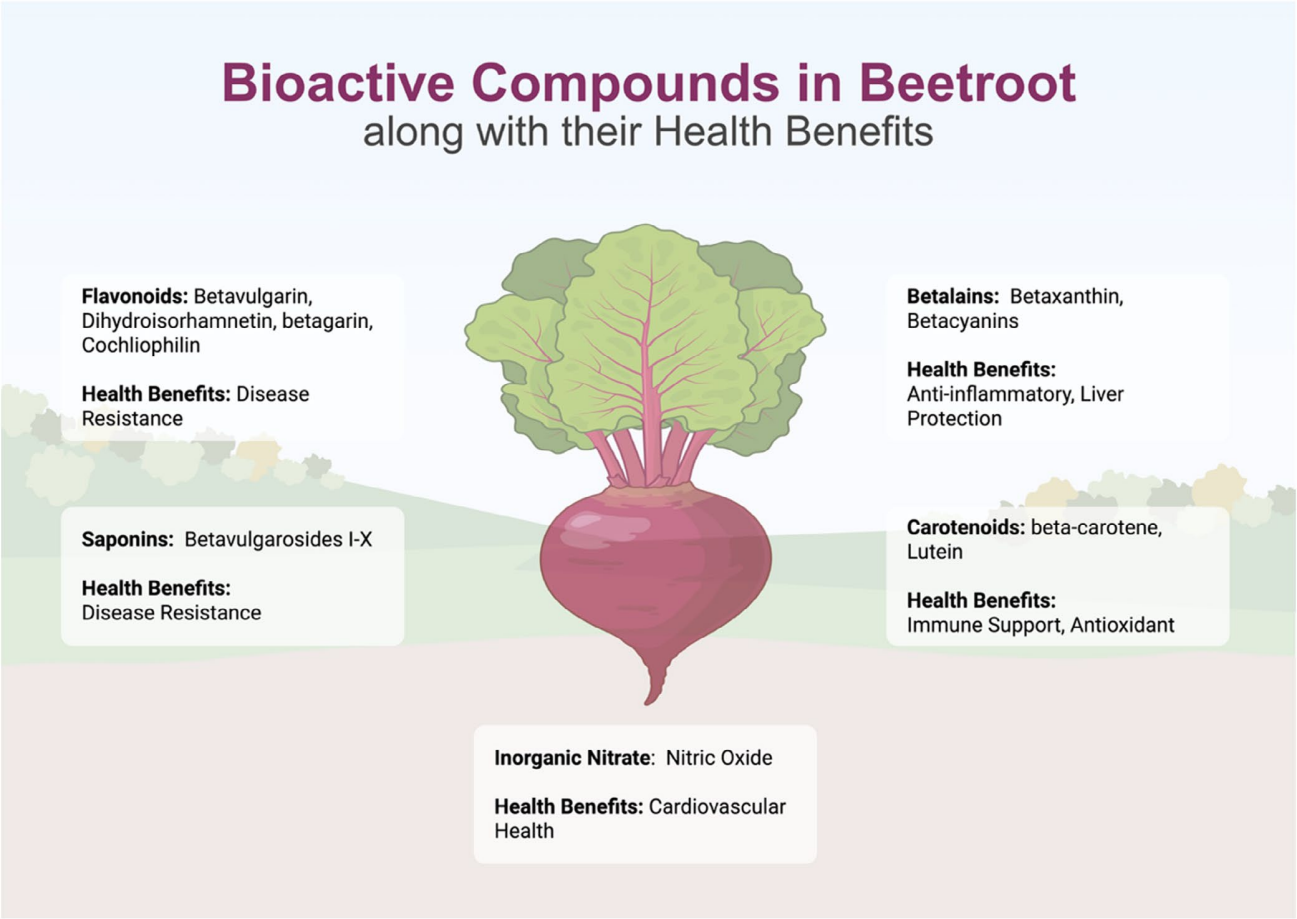
Bioactive active compounds in beetroot.

Beetroot comprises a high concentration of many substances that are beneficial to human health and may help prevent disease. Beetroot is an excellent source of diverse phytochemicals, including ascorbate, carotenoids, flavonoids, polyphenols, saponins, and high levels of nitrates as shown in Figure 3. In addition, it provides a rich supply of dietary fiber, further enhancing its nutritional value (Bangar et al. 2022).

Some additional biologically active compounds identified in beetroot include alkaloids such as calystocin B1, calystocin B2, ipomine, and calystocin C1, along with both essential and nonessential amino acids, including methionine, cysteine, leucine, isoleucine, histidine, arginine, and others (Milton‐Laskibar et al. 2021).

### 7.1 Inorganic Nitrate

Beetroots have a level of inorganic nitrate that is estimated to be 250 mg per kilogram of their fresh weight. It is assumed that the in vivo conversion of nitrates to nitric oxide (NO), not mediated by any particular physiological activity, causes the beneficial effect of nitrates (Mirmiran et al. 2020).

Nitric oxide is a multifunctional messenger molecule that plays a significant role in both the cardiovascular system and the metabolic system. Nitric oxide is a molecule that is typically produced in the endothelium and has the potential to have a noteworthy influence on the regulation of vasculature balance, either as a result of its powerful vasodilator effect, its ability to regulate systemic blood pressure, or its ability to delay the process of atherogenesis (Vasconcellos et al. 2016).

### 7.2 Flavanoids

The bioactive flavonoids can have many positive impacts on human health, such as serving as antioxidants. Beetroot contains four major types of flavonoids: betavulgarin, betagarin, cochliophilin A, and dihydroisorhamnetin. Additional flavonoid compounds isolated from beetroot include 2,5‐dihydroxy‐6,7‐methylenedioxyisoflavone, 3,5‐dihydroxy‐6,7‐methylenedioxyflavanone, and 5‐hydroxy‐6,7‐methylenedioxyflavone (Tan and Hamid 2021).

### 7.3 Saponins

Plants defend themselves against herbivores and diseases through the production of saponins, which are bioactive molecules. Initial studies revealed eleven unique triterpene saponins in beetroot. Oleanolic acid derivatives were present in each of the saponins. According to Mroczek et al. (2019), the saponins betavulgarosides I–VIII were identified in beetroot, while betavulgarosides I–V, IX, and X were detected in the leaves.

### 7.4 Carotenoids

The phytonutrients carotenoids are accountable for the color of fruits and vegetables. They are also important when it comes to defending the body against diseases, some of which may be life‐threatening. Beetroot has carotenoids, which not only activate the immune system but also serve as antioxidants and anticarcinogens. According to Upadhyay (2018), beetroot leaves contain beta‐carotene and oxygenated derivatives known as xanthophylls, including lutein.

### 7.5 Betalains

In addition, betalains are a type of highly bioactive pigment present exclusively in beetroot, and thus one of the few vegetables that have them. Beetroot has such pigments. Two types of betalain pigments predominate: “betacyanin” pigments, which are reddish‐violet in color, and “betaxanthin” pigments, which are yellowish‐orange in color (Nikan and Manayi 2019). Natural food and beverage colorants of red beetroot betalain extract, mainly betanin, are utilized in various meals and beverages. Aside from their red hue, betalains have numerous beneficial biological actions (Masih et al. 2019).

Some of their functions include the prevention of cancer, inflammation, oxidative stress, and liver injury. Scientists have found that betalains are very bioavailable to humans. Apart from maintaining most of their antioxidant activity, betalains are stable during the digestive process. Due to this, betalains are gaining more importance as food additives that enhance health (Hadipour et al. 2020).

### 7.6 Betacyanins and Betaxanthins

All betalains fall into either betaxanthins, which are yellow, or betacyanins, which are reddish‐violet in color. Beetroots contain betacyanins at concentrations ranging from 75% to 95%. Betacyanins comprise around 80% of pigments in beetroot red in color. These betacyanins include betanin and its isomer, isobetanin (Leong et al. 2018).

Vegetables typically exhibit a higher concentration of pigments on their surface, with levels decreasing toward the inner flesh. Previous studies on processed beetroot juice have shown that betanin, present at 300–600 mg/kg, is the predominant betalain, followed by vulgaxanthin and isobetanin (Sadowska‐Bartosz and Bartosz 2021). Yellow‐orange betaxanthins, which make up 5%–25% of total betalains, are formed through the condensation of amino acids or biogenic amines with betalamic acid. These betalains remain stable within a pH range of 3–7. Within the pH range of 3–7, the betalains are able to maintain their stability. There have been some studies that have suggested that betaxanthins and betacyanins are also able to absorb visible light; nevertheless, the disparity in structure between these composites impacts their capability to absorb light (Skalicky et al. 2020).

## 8 Health Benefits of Beetroots

Intake of beetroot has been linked to a diversity of health advantages, comprising anticancer activity, the prevention of cell damage, improvements in digestive health, and enhanced performance in physical exercise. Table 3 summarizes the various health benefits associated with beetroot consumption. Beetroot has also been found to support pregnancy due to its high folic acid content, while its carotenoids contribute to improved blood flow and vision (Chen et al. 2021).

**TABLE 3:** Health benefits of beetroots.

Health benefit	Description	Keywords	References
Antioxidant potential	Beetroot antioxidants like betalains help reduce oxidative stress	Antioxidants, betalains, oxidative stress	Georgiev et al. (2010), Krajka‐Kuźniak et al. (2013), Chen et al. (2021)
Cardiovascular health	Nitrates in beetroot support heart health and lower blood pressure	Nitrates, blood pressure, heart health	Bahrami et al. (2021), Mirmiran et al. (2020), Li et al. (2016)
Anti‐inflammatory effects	Betalains help lower inflammation and related markers	Anti‐inflammatory, cytokines, betalains	Hadipour et al. (2020), Bangar et al. (2022)
Protection against cancers	Compounds like betanin may help prevent cancer by inhibiting tumor growth	Cancer, betanin, tumor inhibition	Tan and Hamid (2021), Kapadia et al. (2011), Lechner et al. (2010)
Enhancement of cognitive ability	Nitrates improve brain blood flow and cognitive function	Cognition, brain, blood flow	Kelly et al. (2013), Kennedy (2016)
Improved athletic performance	Beetroot boosts endurance and oxygen use in muscles	Performance, endurance, nitrates	Peeling et al. (2015), Clifford et al. (2015), Oskarsson and McGawley (2018)
Liver protection	Beetroot supports liver detox and protects against damage	Liver, detox, antioxidants	Krajka‐Kuźniak et al. (2012), Nwaogwugwu et al. (2019)
Kidney protection	Helps protect kidneys from oxidative and chemical damage	Kidney, detox, oxidative stress	Albrahim (2020), Cosola et al. (2018)
Folate‐linked benefits	Folate in beetroot aids DNA repair and prevents birth defects	Folate, DNA, neural tube defects	Fenech (2012), Devalia et al. (2014), Bibbins‐Domingo et al. (2017)
Anti‐fatigue properties	Supports energy metabolism and reduces fatigue, including in cancer patients	Fatigue, energy, beetroot	Swartz et al. (2019)
Antimicrobial effects	Shows antibacterial activity against some pathogens	Antimicrobial, phytochemicals, bacteria	Coy‐Barrera (2020), Pandita et al. (2020)
Genomic stability	Folate helps maintain DNA stability and reduce mutation risk	Genomic stability, folate, DNA	LeBlanc et al. (2018), Catala et al. (2019)
Metabolic syndrome management	Improves cholesterol, blood sugar, and insulin response	Metabolism, insulin, lipids	Mirmiran et al. (2020), Milton‐Laskibar et al. (2021)
Support in chemotherapy	Betanin may boost chemotherapy effects and reduce side effects	Chemotherapy, adjuvant, betanin	Hijaz et al. (2016), Tan and Hamid (2021)
Antiaging effects	Antioxidants help reduce signs of aging and cell damage	Antiaging, antioxidants, cell health	Masih et al. (2019), Ravimannan and Nisansala (2017)

### 8.1 Antioxidant Potential

It is generally accepted that the biological atmosphere of a cell is in a state of redox balance when its metabolic processes are functioning normally. In other words, there is a balance between oxidizing and reducing substances. Reactive oxygen species and reactive nitrogen species are common names for molecules that are capable of oxidation. These radicals are continuously created in the metabolic processes of cells. However, the cell's antioxidant defenses can be overpowered by high levels of exposure to these species, leading to imbalanced redox homeostasis. This instability in redox homeostasis is what gives rise to the state that is often known as oxidative stress (Ravimannan and Nisansala 2017).

The beetroot is a tremendously ironic source of numerous different types of antioxidant chemicals. Betalains, which are found in beets, boost the body's resistance to oxidation, which in turn enriches metmyoglobin and low‐density lipoproteins in humans (Bangar et al. 2022). Betalains and other phenolic compounds can help treat inflammation in joints, bones, and blood vessels, in addition to preventing oxidative damage to lipids. Betalains protect lipids from damage caused by free radicals. This restoration is beneficial for patients with asthma and osteoporosis, particularly in conditions associated with inflammation. Because of this, betalains were proven to show anti‐inflammatory and antiradical actions, which contribute to the regulation of oxidative stress‐related illnesses in human beings (Chhikara et al. 2019).

### 8.2 Cardiovascular Health

Ingestion of beetroot, which is a source of nitrate, offers a natural method of boosting the amount of nitric oxide that is available in vivo. This has the potential to prevent and manage hypertension while also enhancing endothelial function. A thorough analysis of the experimental results indicated that nitrate is a positive vascular endothelial effector. This indicates that it promotes vasodilation and reduces blood pressure in individuals with both normal and elevated blood pressure (Clifford et al. 2015).

Recently, Baião et al. (2020) reported that dietary nitrate from beetroot, along with its bioactive compounds, exerts significant cardioprotective effects in both healthy and hypertensive individuals. Beetroot nitrate and nitrate‐derived nitrite enhance nitric oxide synthesis, improving endothelial function, reducing arterial stiffness, and lowering blood pressure, while phytochemicals like betanin, saponins, polyphenols, and organic acids help limit oxidative stress and modulate gene expression. Overall, these findings indicate that beetroot nitrate and its phytochemicals may serve as an effective supportive therapy for cardiovascular disease, with formulations that can be tailored for either short‐term or long‐term use. Another research examination by Bahrami et al. (2021) explored the prospective benefits of supplementation with inorganic nitrate in beetroots for adult cardiovascular risk factors. The study suggests that inorganic nitrate present in beetroot may improve cardiovascular function.

### 8.3 Anti‐Inflammatory Effects

Under typical conditions, inflammation is considered to be a good process because it governs our inherent reaction to biological and physiological stressors like injury, infection, as well as other pathogens that have the potential to damage the body and disturb homeostasis. Chronic inflammation, which can lead to long‐term cell malfunction if it is not properly treated, can be caused if the invading material is not removed and normal immune function is not restored. Inflammation that persists over time is frequently thought to play a role in the growth and progression of a number of clinical conditions, including obesity, liver disease, cancer, and cardiovascular disease (Coy‐Barrera 2020).

Since the 1970s, nonsteroidal anti‐inflammatory medications have been the standard treatment for inflammatory illnesses. In spite of this, the focus has recently switched toward the anti‐inflammatory properties of natural resources and their prospective usage as replacement for synthetic treatments. This is because of the harmful implications and bad side effects that these synthetic medicines have on health. Betalains and extracts of beetroot have recently been discovered to be powerful anti‐inflammatory agents. By dealing with pro‐inflammatory signaling cascades, it appears to be the mechanism that underlies at least a portion of their anti‐inflammatory actions. In addition, it has been established that betalains significantly inhibit the expression of cyclooxygenase‐2, an essential molecule that assists as a precursor for the production of the proinflammatory metabolites of arachidonic acid called prostaglandins (Georgiev et al. 2010).

### 8.4 Protection Against Cancers

There is increasing evidence that chronic inflammatory responses are related to the development of malignant tumors, and betalain extracts resultant from beets may be able to counter this effect. In vivo randomized controlled studies, rats treated with beetroot showed significantly fewer inflammatory lymphocytes in esophageal tumors. Additionally, it has been demonstrated that betacyanin isolates have chemopreventive effects on human prostatic, skin, breast, and pancreatic cancer cells, as well as lung, skin, and liver cancer cells in animal models (Swartz et al. 2019).

According to Kapadia et al. (2011), red beetroot extract exhibits anticancer properties. Red beetroot extract was found to be cytotoxic to androgen receptors in prostate cancer cells, indicating its potential as an anticancer agent capable of reducing treatment‐related toxicities. Another investigation by Lechner et al. (2010) showed that rat esophagus tumors induced by N‐nitroso dimethylbenzylamine can be suppressed with red beetroot food color. The dye reduced cell proliferation, inflammation, and stimulated apoptosis, and consumption in drinking water did not cause overt toxicity.

### 8.5 Enhancement of Cognitive Ability

It has long been known that nitric oxide insufficiency is linked to cognitive impairment. Beetroot is becoming more well‐known as a source of nitric oxide. The alteration in the cerebral prefrontal cortex blood flow “CBF” caused by nitrate comprising beets was linked to improved cognitive function. Similarly, supplementation with beets high in nitrate has been shown to boost regional CBF and consequently improve cognitive function in the elderly (Wightman et al. 2015).

The investigation was led by Kelly et al. (2013) on 12 healthy older adults who received nitrate‐rich concentrated beetroot juice or a nitrate‐depleted beetroot juice placebo. It caused a rise in plasma nitrite levels, reduced systolic and diastolic blood pressure, and increased walking response time on the treadmill. Nevertheless, it did not improve the ability to walk or brain function. Similarly, Wightman et al. (2015) have shown that the parameters of the prefrontal cortex are altered. Cerebral blood flow, or CBF, caused by nitrate‐containing beetroots is connected to the improvement of cognitive function.

### 8.6 Improved Athletic Performance

In recent years, there has been a surge in curiosity in several nutrients for boosting physical activity performance. Due to the positive effects of dietary nitrates on athletic performance, nitrate‐rich beetroot has attracted considerable attention. Dietary nitrate is converted to nitrite and then to nitric oxide and other nitrogen intermediates, which play a role in enhancing athletic performance (Stanaway et al. 2017).

Regarding the influence of beet on the exercise routine of various subjects, multiple studies have been conducted. A study in male and female athletes demonstrated that beetroot juice supplementation enhances kayaking performance by improving aerobic efficiency during endurance tasks, resulting in a 1.7% improvement in the 500‐m time trial (Peeling et al. 2015).

Another investigation led by Oskarsson and McGawley (2018), assessed the effect of caffeine and beet juice supplementation on treadmill running performance in seven males and two females. It was concluded that there were no noteworthy differences in oxygen uptake, running efficiency, respiratory exchange ratio, heart rate, or perceived exertion.

### 8.7 Protection From Liver and Kidney Diseases

Beetroots contain nutrients and vitamins that help to remove toxins from the blood and liver, as well as the ability to treat digestive, liver, and kidney issues, in particular the formation of hepatic fat plaques caused by alcohol addiction, protein deficiency, or diabetes (Cosola et al. 2018).

In previous studies conducted by Albrahim (2020), the kidney‐protective properties of *B. vulgaris* juice (RBR) and silver nanoparticles (AgNPs) were investigated in male rats. However, in this study, it was concluded that RBR's posttreatment reversed abnormalities and enhanced kidney function indicators. According to a study examining the activation and gene expression effects of betanin on hepatic cell lines, Krajka‐Kuźniak et al. (2013) showed that beetroot juice protects from liver damage by triggering the Nrf2 antioxidant response element pathway.

Similarly, Krajka‐Kuźniak et al. (2012) demonstrated that when NDEA (N‐nitrosodiethylamine) is used in combination with red beetroot rich in antioxidants, it reduces enzyme markers, increases CYP2B activity, and prevents DNA damage and liver disease biomarkers.

### 8.8 Other Therapeutic Effects

Beetroot is thought to be a possible therapeutic treatment for several clinical conditions linked to oxidative stress and inflammatory processes. Red beet consumption may also aid in protecting against age‐related ailments. Beets are abundant in ascorbic acid and fiber, as well as manganese and potassium, which are important nutrients (Clifford et al. 2015).

Potassium is necessary for normal muscle and nerve functioning, as it reduces the use of ATP, the body's energy source, by muscles. Fiber aids in the movement of wastes throughout the intestines and prevents constipation. Meanwhile, the antioxidant compounds in beets offer protective effects against colon cancer. Beetroot is traditionally believed to have therapeutic properties, including carminative, emmenagogue, hemostatic, and kidney‐protective effects (Nwaogwugwu et al. 2019).

## 9 Use of Beetroot in the Development of Functional Foods

Beetroot is rummage‐sale as an element in a diversity of foods. These products have a positive influence on human health and provide an opportunity for the development of different functional foodstuffs (Chhikara et al. 2019).

As part of a healthy lifestyle and diet, functional foods have become increasingly accepted. Different fractions of beets may be produced, e.g., powder, peels, extracts, pulps, pomace, or juice, for use as ingredients in foodstuffs that are enriched with a number of beetroot fractions. Noodles, cookies, bread, biscuits, cakes, muffins, pasta, extruded snacks, yogurt, butter, curd, ice cream, cream cheese, paneer, milk, and fermented beverages are a few examples of these functional food products because of the wide range of nutrients they contain, including folic acid, vitamin C, potassium, manganese, iron, and fiber (Punia Bangar et al. 2022).

## 10 Conclusion and Future Perspectives

Folate, also recognized as vitamin B9 or folacin, is a water‐soluble B‐vitamin found in both natural and enriched foods. For its physiological function, it relies on the conversion of folates that cannot be produced by the human body. The dietary requirement was originally based on the amount necessary in order to prevent severe shortages of essential nutrients, such as anemia. Folate cofactors are essential for bodily functions, but imbalances and unhealthy lifestyles can lead to health issues like fatty liver disease, cancer, and congenital disabilities.

Folate deficiency can lead to chronic disorders like heart disease and cognitive impairment. Beetroot, a rich source of nutrients, has antioxidant properties that may protect against colon cancer, hemostatic and renal protection. In order to fully elucidate how beetroot‐contracted folate and allied phytochemicals contribute to DNA synthesis, repair, and methylation, future research into beetroot's shield against folic acid deficiency should focus on precise biochemical mechanisms. To determine whether it prevents folate‐associated diseases including neural tube defects, megaloblastic anemia, and cardiovascular disease, clinical tests must be conducted. Beetroot juice, powder, or extract can be maximized for specific nutritional therapy using bioavailability studies. Public health programs incorporating beetroot as a natural dietary source of folate may offer a long‐term, plant‐based alternative to synthetic folic acid supplements, especially in regions where the deficiency is prevalent.

## Author Contributions

**Amara Rasheed:** writing – original draft (equal). **Farhan Saeed:** methodology (equal). **Ali Ikram:** supervision (equal). **Muhammad Tayyab Arshad:** writing – review and editing (equal). **Sammra Maqsood:** data curation (equal). **Feroza Naveed:** visualization (equal). **Mariam Islam:** formal analysis (equal). **Emmanuel Laryea:** validation (equal).

## Funding

The authors have nothing to report.

## Ethics Statement

This study did not involve humans or animals.

## Consent

This study did not involve humans.

## Conflicts of Interest

The authors declare no conflicts of interest.

## Data Availability

The data that support the findings of this study are available from the corresponding author upon reasonable request.
